# The efficacy of positioning stents in preventing Oral complications after head and neck radiotherapy: a systematic literature review

**DOI:** 10.1186/s13014-020-01536-0

**Published:** 2020-04-28

**Authors:** Dong Chen, Xiaoju Chen, Xinmei Chen, Nanchuan Jiang, Li Jiang

**Affiliations:** 1 State Key Laboratory of Oral Diseases & National Clinical Research Center for Oral Diseases, NO.14, 3rd Section of Ren Min Nan Rd., Chengdu, 610041 Sichuan China; 2 Department of Endodontic Dentistry, Sichuan University West China Hospital of Stomatology, NO.14, 3rd Section of Ren Min Nan Rd., Chengdu, 610041 Sichuan China; 3 Department of Radiology, Union Hospital, Tongji Medical College, Huazhong University of Science and Technology, 1277 Jiefang Avenue, Wuhan, 430022 Hubei China

**Keywords:** Head and neck neoplasm, Radiotherapy, Stents, Complications, Systematic review

## Abstract

**Background:**

Positioning stent in head and neck radiotherapy seems to have benefit to prevent oral complications but it hasn’t been summarized by an evidence-based method.

**Objectives:**

This review aims to evaluate the efficacy of positioning stents in preventing oral complications after radiotherapy.

**Methods:**

We conducted an electronic search in MEDLINE, EMBASE, Cochrane CDSR, and Cochrane CENTRAL database for randomized-controlled clinical trials, controlled clinical trials and cohort studies that assessed oral complications after head and neck radiotherapy with positioning stents. Two reviewers extracted information on radiotherapy, follow-up period, oral complications and assessments independently.

**Results:**

Three RCTs and two cohort studies were included in this review. Oral complications such as mucositis, xerostomia, taste alteration, trismus, salivary changes, dysphagia and pain on swallowing were assessed by different methods in these studies.

**Conclusions:**

Oral complications were common in patients after head and neck radiotherapy. There is insufficient evidence that positioning stents have a preventive effect against xerostomia, and it needs more high-quality and prospective trials with long-term follow-up to support it.

## Introduction

Although radiotherapy is an effective treatment for head and neck cancer (HNC), it would bring acute and long-term adverse effects. Oral complications are very common in patients treated with radiotherapy, for example, mucositis, xerostomia, taste disorders and dysphagia. Furthermore, radiation caries, dentition defect and trismus may increase the cost of dental treatment and management. These complications may have a considerable impact on patients’ quality of life (QoL). Modern radiotherapy technique like intensity modulated radiotherapy (IMRT) can deliver a lower dose to normal tissue therefore decreasing the risk of suffering oral complications [1]. However, it is not available in poor region on account of the high cost. Additionally, salivary gland transfer is probably effective to prevent oral complications like xerostomia, whereas it requires specialized training to perform the surgical operation [2, 3].

Intraoral stent is another potential way to help reduce oral complication rate. It is a kind of individualized intraoral device worn by patients during radiation therapy, which can protect adjacent organ at risk (OAR), such as parotid glands, submandibular glands, tongue, swallowing structures and oral mucosa. The patients can wear it like a removable denture when undergoing radiotherapy. There are two types of stents [4]: The one is positioning stent, an open mouth device to ensure reproducible position of the mandible and spare normal tissue like salivary glands, tongue and part of oral mucosa from radiation target volume; Another is shielding stent, made of shielding materials therefore it can directly block electron beams. There are some difference between them in fabrication and working condition. Herein we mainly discuss about positioning stent below and shielding stent is beyond the scope of this article.

Pilot studies [5–8] have investigated the fabrication and utility of intraoral stents. It wouldn’t spend much time and money to fabricate a high-precision intraoral stent [5, 7, 8]. additionally, they help reduce the radiation dose of OAR, with fewer complications reported by patients [6, 7]. Furthermore, it is also reported that positioning stent could reduce setup errors in IMRT for head and neck cancers [9]. However, to the best of our knowledge, the efficacy of positioning stents has not been sufficiently summarized by an evidence-based method. Therefore, the objective of this study is to review literatures systematically and evaluate the positioning stents in decreasing the risk of oral complications after radiotherapy.

## Methods

### protocol

The reporting of this systematic review was presented following the Preferred Reporting Items for Systematic Reviews and Meta-Analyses (PRISMA) statement [10] whenever possible. The PICOS principle (participants, interventions, controls, outcomes, and study designs) was applied during the investigation. Specifically, “Participants” included HNC patients undergoing radiotherapy. “Interventions” were intraoral stents (positioning stents) during radiotherapy. “Controls” were patients without stents undergoing radiotherapy. “Outcomes” were preventions of oral complications, according to specific complication and assessment. “Study designs” included randomized-controlled clinical trials (RCTs), controlled clinical trials (CCTs) and cohort studies. No language or time restrictions were imposed.

### search strategy

We conducted an electronic literature search using PubMed, EMBASE, Cochrane Database of Systematic Reviews (CDSR), and Cochrane Central Register of Controlled Trials (CENTRAL) databases in January 2019. For example, the detailed search strategy for PubMed was presented in Table 1. In addition, we also consulted the ongoing clinical studies on the ClinicalTrials.gov and gray literatures on the System for Information on Gray Literature in Europe database (SIGLE). The electronic search was complemented by a hand search of bibliographies from full-text articles and related reviews.

**Table 1:** Detailed search strategy for PubMed

#1	((((“Head and Neck Neoplasms”[Mesh])) OR (Head and Neck Cancer*)) OR (Head and Neck tumor*)) OR (Head and Neck tumour)
#2	((“Radiotherapy”[Mesh]) OR radiotherapy*) OR radiation therapy*
#3	(“Stents”[Mesh]) OR intraoral stent*
#4	#1 AND #2 AND #3

### study selection

Two reviewers (Dong C, Xiaoju C) conducted the study selection in duplicate independently, and any disagreement was solved in consensus via discussion or by a third reviewer. The screening of titles and abstracts was performed and studies that were not related to intraoral stent or did not meet the inclusion criteria were excluded. Then, the full texts of the qualified articles were obtained for independent data collection and quality assessment. The inclusion and exclusion criteria are as follows:

#### Inclusion criteria

1. RCTs, CCTs, and prospective or retrospective cohort studies.
2. Studies with quantitative clinical outcomes of oral complications (subjective or objective).
3. The details of the materials and methods should be reported.

#### Exclusion criteria

1. In vitro studies, case reports, and case series.
2. Stent designs are shielding stent.
3. Clinical outcomes are radiation dosimetric analysis or CT simulations only.

### data extraction

The data were independently extracted by two reviewers using a designed data collection list in duplicate, and checked for agreement. The list included the study design, sample size and characteristic, radiotherapy, follow-up period and oral complications. Clinical outcomes were also collected, which included objective or subjective assessments of specific oral compilations.

### quality (bias) assessment

The bias assessment of the selected RCTs was performed according to the Cochrane Collaboration tool for assessing risk of bias [11]. The bias and quality of the non-randomized studies were evaluated by the ROBINS-I tool (Risk Of Bias In Non-randomized Studies–of Interventions) [12].

## Results

### included studies

**Fig. 1:**
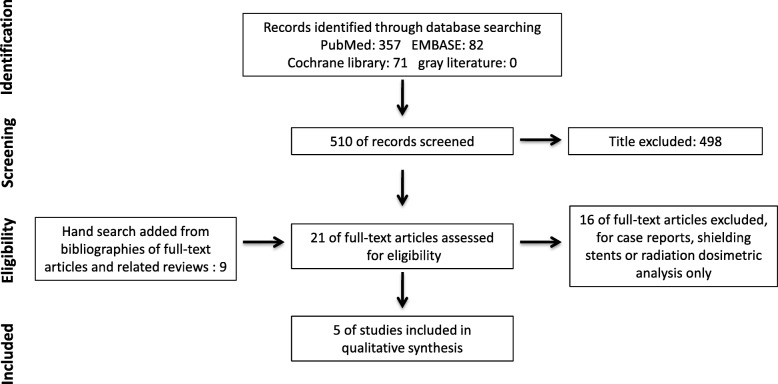
The flow diagram for selection of studies

A total of five studies [13–17] were included in this systematic review. The process for selecting studies is presented in Fig. 1. The methodological and patient characteristics of the included studies are shown in Table 2. There were three RCTs and two retrospective cohort studies included, totally 101 patients with positioning stents and 110 patients in control group. The follow-up period is relatively short because five studies lasted about 2 months, and only in one study the patients were followed up for 6 months. Tumors were located in mandible, floor of mouth, tongue, lip, buccal region, maxilla and nasopharynx in these patients. Radiotherapies were IMRT or conventional radiotherapy (CRT). Among these studies, several oral complications were investigated and the methods to assess oral complications were different. Salivary flow rate and QoL questionnaire were methods used to assess xerostomia. Whereas mucositis severity was assessed by using grading tools like National Cancer Institute Common Toxicity Criteria Version 2.0 (CTC 2.0) and the classification criteria of the World Health Organization. Taste alteration and the maintenance of mouth opening were assessed by a taste test and measuring the maximal mouth opening respectively. One study employed Radiation Therapy Oncology Group (RTOG) 045 Head and Neck adverse effects grading tool to investigate radiotoxicity which covered dental caries, mucositis, dry mouth, salivary changes, taste alteration, dysphagia, trismus and pain on swallowing. Radiation dosage was also examined and analyzed in certain studies. Evidence of long-term complications is insufficiency on account of short follow-up period, therefore the results only reveal short-term protective effect of positioning stent.

**Table 2:** Methodological and patient characteristics of the included studies

Study	Year	Study design	Tumor location	Radiotherapy	Sample size^a^	follow-up time	Drop out	Oral complications	Assessment tools
Mall et al. [17]	2016	RCT	Carcinoma of the posterior tongue	CRT	15/15	6 months	3/3	Xerostomia	(QLQ-H&N35) questionnaires and salivary flow rate
Nayar et al. [15]	2016	retrospective cohort study	Cancer on/near the mandible or maxilla	IMRT, CRT	24/33	2 months	0	Trismus	The maximal mouth opening
Verrone et al. [14]	2014	retrospective cohort study	SCC of the tongue and the floor of the mouth	IMRT	19/14	7 weeks	0	Mucositis	Mucositis severity (scored by the classification criteria of the World Health Organization)
Goel et al. [16]	2010	RCT	Posterior third of the tongue cancer	CRT	24/24	60 days	0	Dental caries, mucositis, dry mouth, salivary changes, taste alteration, dysphagia, trismus, and pain on swallowing	The Radiation Therapy Oncology Group’s 045 head and neck cancer adverse events grading tool
Qin et al. [13]	2007	RCT	Nasopharyngeal carcinoma	CRT	19/24	8 weeks	0	taste alteration, mucositis	Taste test and mucositis grade (National Cancer Institute Common Toxicity Criteria Version 2.0)

^a^sample size: study group size/control group size;

The bias assessment of the included studies is displayed in Table 3 and Table 4. Both two cohort studies were considered to have serious risk of bias, according to the ROBINS-I risk of bias assessment. One RCT was considered to have high risk of bias due to a high dropout rate. The others were judged to have an unclear risk of bias.

**Table 3:** Cochrane Collaboration tool risk of bias assessment

Study	Random sequence generation	Allocation concealment	Blinding of participants and personnel	Blinding of outcome assessment	Data integrity	Selective reporting	Other sources of bias	Risk of bias
Mall et al. [17]	Low risk	Low risk	High risk	Low risk	High risk	Low risk	Low risk	High
Goel et al. [16]	Unclear risk	Unclear risk	Unclear risk	Unclear risk	Low risk	Low risk	Low risk	Unclear
Qin et al. [13]	Unclear risk	Unclear risk	Unclear risk	Unclear risk	Low risk	Low risk	Low risk	Unclear

**Table 4:** ROBINS-I risk of bias assessment

Study	Confounding	Selection of participants into study	Classification of intervention	Deviation from intended intervention	Missing data	Measurement of outcomes	Selection of the reported results	Over all
Nayar et al. [15]	Serious risk	Low risk	Low risk	Low risk	Low risk	Low risk	Moderate risk	Serious risk
Verrone et al. [14]	Serious risk	Low risk	Low risk	Low risk	Low risk	Moderate risk	Moderate risk	Serious risk

### efficacy of positioning stents in CRT

Conventional radiotherapy delivers external beam radiation without fitting the radiation beams close to the target volume. In these studies that involved with CRT, xerostomia, taste alteration, mucositis and other radiotoxicities were researched.

Xerostomia reflects the damage to salivary glands. It can be investigated objectively like salivary flow rate measurement or subjectively like questionnaire. Mall et al. [17] reported salivary flow rates dropped both in study group and control group after radiotherapy, but study group reserved higher unstimulated salivary flow rates than control group in 3-month (*P* = 0.044) and 6-month (*P* = 0.023) intervals, as same as stimulated salivary flow rates in 3-month (*P* = 0.021) and 6-month (*P* = 0.020) intervals. A simultaneous QoL questionnaire revealed similar change to support it.

Qin et al. [13] designed a taste test in which patients degust and distinguish different concentrations of solution (viz. sugar, salt, berberine, and acetum) to analyze taste alteration. Control group showed significantly higher occurrence rate of taste disorder than study group (*P* = 0.000) 8 weeks after radiotherapy. A dosage simulation of one patient showed a lower radiation dose to the tongue when waring positioning stent. The authors also investigated the severity of mucositis using CTC 2.0 tool but received non-significant difference between two groups (*P* = 0.47).

Goel et al. [16] investigated several oral complications using Radiation Therapy Oncology Group (RTOG) 045 Head and Neck adverse effects grading tool. Specifically speaking, dental caries, mucositis, dry mouth, salivary changes, taste alteration, dysphagia, trismus, and pain on swallowing were subjectively graded 30, 45 and 60 days after radiotherapy. No dental caries was observed in all the patients, possibly because of the short follow-up period. Patient-reported symptoms related to mucositis and dry mouth were milder in stent group, with statistical significance (*P* < 0.01, *P* = 0.06 respectively). But it should be noted that the “mucositis” was limited as palate mucositis in radiotherapy of tongue cancer according to the paper. Other complications were found no significant difference between two groups.

Overall, risk of xerostomia was reported decreased when positioning stents were applied in both objective and subjective measurements. Findings about mucositis were inconsistent, as assessment method, criterion and time were different.

### efficacy of positioning stents in IMRT

IMRT is a great progress in radiation delivering. But this treatment requires highly repeatable position to ensure consistent radiation dosimetry. Usually a thermoplastic mask is applied to immobilize the head. Positioning stent is compatible with thermoplastic mask in IMRT and enables clinicians to achieve more precise position while sparing OARs from damage. Two studies evaluated the efficacy of positioning stents in IMRT.

Nayar et al. [15] measured maximal mouth opening before and 1–2 months after radiotherapy, and found a significantly better mouth opening in study group (*P* < 0.01). Patients in study group almost maintained their mouth opening while patients without stent lost about 1 cm maximal mouth opening. Besides, the radiation dosages to the jaws were compared in two groups. A significant dose reduction to the maxilla was observed in tumors of the mandible with a stent (*P* < 0.01). They also found there were some cases that showed little dose reduction to the opposing jaw, considering that the tumors involved structures near both jaws and the target volumes were large.

In another study conducted by Verrone et al. [14], the investigators classified mucositis by symptoms according to classification criteria of the World Health Organization, after the IMRT for treating cancers located in the tongue and floor of the mouth. There was a tendency in delaying the occurrence of severe mucositis in patients with a stent 3 to 4 weeks after radiotherapy, but there was little statistical significance (*P* = 0.82). In the dosimetric analysis, the average dose to the maxilla and ipsilateral parotid was lower with a stent (*P* = 0.05). No significant difference was observed when comparing the mean dose of the mandible and the contralateral parotid glands.

These two studies on efficacy of positioning stents in IMRT revealed that the dose to adjacent OARs has been reduced. As for oral complications, mucositis cannot be effectively prevented. Whether the risk of suffering from trismus could be reduced stayed unclear. Although maximal mouth opening decreased in 2 months, long term follow-ups are needed to track the incidence of trismus.

## Discussion

### The mechanism of positioning stents to prevent complications

The core mechanism of positioning stents is sparing OARs, usually glands and mucosa around maxilla or mandible, from radiation field, thus preventing complications [4]. A vertical jaw position of one-half to three-quarter of maximum mouth opening is immobilized. And impression materials are applied to record the occlusion, so the position is repeatable during treatment. In two included studies [13, 15], tumors were located above maxilla. With a large mouth opening position, glands and mucosa around the mandible are spared away from radiation field. Some kinds of positioning stents are also designed with a horizontally extended plate that could depress the tongue [7, 16, 17]. As a result, the radiation dose to these organs is reduced. When the tumor is located in mandible or around, the sparing area of position stents is upper jaw, because the radiation field is pushed downward. Palate, maxilla, temporomandibular joint, etc. are protected. Specifically, in the studies conducted by Verrone et al. [14] and Nayar et al. [15], mean dose to the maxilla is reduced. Meanwhile, the patients also have to open their mouth to insert and keep the stent in. The stent can serve as a mouth open training device in this condition and may help them maintain mouth opening [15].

The risks of oral complications are associated with sparing areas of OARs. Salivary glands are sensitive to radiation, but they are widespread in oral tissue so a positioning stent could retain salivary secretion function partly to decrease the risk of xerostomia and salivary changes. Oral mucosae are also spared from radiation, and potentially receive a lower dose. But considering the sensitivity and wide spreading, mucositis degree would decline in a limited range. Thus the included studies (Qin et al. [13] and Verrone et al. [14]) found weak effects in preventing mucositis. Goel et al. [16] reported milder mucositis in palate, but we suppose that when considering floor of the mouth or togue, the outcome might be different. Taste alteration is usually associated with reduced salivary volume and flow, because it impairs the physical contact of foods with the taste papillae [18], and these papillae might be destroyed by radiation [19]. Thus, positioning stents that spare salivary glands and tongue could decrease the risk of taste alteration. Other complications like trismus, dysphagia, and pain on swallowing are caused by a complex of inflammation and fibrosis of muscle and soft tissue, nerve lesion and other damage, which are not clearly explained [20, 21]. Sometimes the protection range of positioning stents could be limited considering the primary tumor location (too close to throat or masticatory muscles).

### Oral complications assessment

Radiotoxicity is a concerned problem and many radiotherapy toxicity criteria or grading systems are performed to assess oral complications. QoL questionnaires and patient-reported symptoms directly reflect pain or uncomfortable caused by radiation. These methods are considered subjective or semi-subjective. Objective measurements are available in specific complications. Salivary flow rate measurement is widely used as an objective way to assess xerostomia, and maximal mouth opening as a sign of trismus. Furthermore, it is recommended to use both assessments to minimize bias. Although the included studies in this review mainly use single method. The heterogeneity in the methods of oral complications assessment also resulted in inability to conduct a meta-analysis.

### Fabrication of positioning stents

Positioning stents are made individualized. And an accurately fabricated stent could ensure patient keeping repeatable position in continuous radiotherapy. This is very important to positioning stents. The predecessors of positioning stents are face-masks or bite blocks, which were immobilizing devices in radiotherapy for head and neck cancers. As dental materials and technology introduced, positioning stents were more convenient and accurate so that they can replace such devices. Impression and model materials were used in the fabrication of positioning stents to record the occlusion. The main body of the stents was constructed based on the model, and usually made of polymer. Positioning accessory then could be inserted. Emerging technology like 3D Printing was also employed recently, making positioning stents fabrication more accurate and convenient [22].

### Limitations

There were several limitations in this systematic review. Firstly, none of the included studies were considered as low risk of bias according to the assessing tool, may resulting in drawing a believable conclusion with a low evidence level. Secondly, because of the heterogeneity in the assessment methods among the included studies, it was difficult to extract the same oral complications assessment data from the clinical outcomes, and this may weaken the results of the review, and we could not conduct a meta-analysis. Thirdly, the total number of participants included was relatively low. Fourthly, the average follow-up period among the included studies was no more than 6 months, while some late complications could arise years after radiotherapy [23]. Nonsignificant difference between two groups in some complications might be due to the short observation time. Hence the result of this review only represents short-term effects.

## Recommendations

### recommendations for clinicians

1. Although for some complications, the benefits of positioning stents have not been sufficiently proved at present, we still recommend using positioning stents as an alternative prevention method in certain case, where clinicians consider specific OARs can benefit from it.
2. Oral complications are common after radiotherapy and have a profound effect on quality of life. Patients should be informed of such risks and taken into oral health management no matter whether prevention was provided.
3. A systematic oral health management before, during and after radiotherapy are better than simple treatments to relieve the symptoms.

### recommendations for researchers

1. We recommend combining objective and subjective methods during oral complications assessment if possible to minimize bias and observe the correlation between the two methods.
2. More high-quality and prospective studies evaluating positioning stents with long follow-up periods are required, because late side effects of radiotherapy are considerable damage to long-term QoL.
3. Mucositis and xerostomia are the most common complications, so they are recommended to be involved when evaluating prevention method of radiotherapy related oral complications.

## Conclusions

Oral complications in HNC patients undergoing radiotherapy were common such as mucositis, xerostomia, taste alteration, trismus. Clinicians designed and fabricated positioning stents, attempting to mitigate the radiation effects. To date, weak evidence has showed their preventive effects against xerostomia. No conclusions could be draw regarding the other oral complications. More high-quality and prospective trials with long follow-up periods are needed to confirm their efficacy.

## Data Availability

All data generated or analyzed during this study are included in this published article.

## References

[1] Van der Veen J, Nuyts S. Can Intensity-Modulated-Radiotherapy Reduce Toxicity in Head and Neck Squamous Cell Carcinoma?. Cancers (Basel). 2017;9(10). 10.3390/cancers9100135. 10.3390/cancers9100135 PMC5664074 28984841

[2] Wu F, Weng S, Li C (2015). Submandibular gland transfer for the prevention of postradiation xerostomia in patients with head and neck cancer: a systematic review and meta-analysis [J]. ORL J Otorhinolaryngol Relat Spec.

[3] Sood AJ, Fox NF, O'Connell BP (2014). Salivary gland transfer to prevent radiation-induced xerostomia: a systematic review and meta-analysis [J]. Oral Oncol.

[4] Kaanders JHAM, Fleming TJ, Ang KK (1992). Devices valuable in head and neck radiotherapy [J]. Int J Radiat Oncol* Biol* Phys.

[5] Johnson B, Sales L, Winston A (2013). Fabrication of customized tongue-displacing stents [J]. J Am Dent Assoc.

[6] Sales LR, Liao J, Johnson B (2011). Customized Tongue-Displacing Dental Stents for Oral Mucosal Sparing and Immobilization in Head and Neck Radiotherapy [J]. Int J Radiat Oncol* Biol* Phys.

[7] Verrone JR, Alves F (2013). D a, Prado J D, et al. impact of intraoral stent on the side effects of radiotherapy for oral cancer [J]. Head & neck.

[8] Wang RR, Olmsted LW (1995). A direct method for fabricating tongue-shielding stent [J]. J Prosthet Dent.

[9] Doi H, Tanooka M, Ishida T (2017). Utility of intraoral stents in external beam radiotherapy for head and neck cancer [J]. Rep Pract Oncol Radiother.

[10] Moher D, Liberati A, Tetzlaff J (2009). Preferred reporting items for systematic reviews and meta-analyses: the PRISMA statement [J]. PLoS Med.

[11] Higgins JPT, Altman DG, Gotzsche PC (2011). The Cochrane Collaboration's tool for assessing risk of bias in randomised trials [J]. BMJ.

[12] Sterne JA, Hernán MA, Reeves BC (2016). ROBINS-I: A tool for assessing risk of bias in non-randomised studies of interventions [J]. BMJ (Clinical research ed.).

[13] Qin W-J, Luo W, Lin S-R (2007). Sparing normal oral tissues with individual dental stent in radiotherapy for primary nasopharyngeal carcinoma patients [J]. Ai zheng = Aizheng = Chinese J Cancer.

[14] Verrone JR, Alves FA, Prado JD (2014). Benefits of an intraoral stent in decreasing the irradiation dose to oral healthy tissue: Dosimetric and clinical features [J]. Oral Surg Oral Med Oral Pathol Oral Radiol.

[15] Nayar S, Brett R, Clayton N (2016). The effect of a radiation positioning stent (RPS) in the reduction of radiation dosage to the opposing jaw and maintenance of mouth opening after radiation therapy [J]. Eur J Prosthodont Restor Dent.

[16] Goel A, Tripathi A, Chand P (2010). Use of positioning stents in lingual carcinoma patients subjected to radiotherapy [J]. Int J Prosthodont.

[17] Mall P, Chand P, Singh BP (2016). Effectiveness of positioning stents in radiation-induced Xerostomia in patients with tongue carcinoma: a randomized controlled trial [J]. Int J Prosthodont.

[18] Rubira CMF, Devides NJ, Ubeda LT (2007). Evaluation of some oral postradiotherapy sequelae in patients treated for head and neck tumors [J]. Brazilian Oral Res.

[19] Deshpande TS, Blanchard P, Wang L (2018). Radiation-related alterations of taste function in patients with head and neck Cancer: a systematic review [J]. Curr Treat Options in Oncol.

[20] Strojan P, Hutcheson KA, Eisbruch A (2017). Treatment of late sequelae after radiotherapy for head and neck cancer [J]. Cancer Treat Rev.

[21] Servagi-Vernat S, Ali D, Roubieu C (2015). Dysphagia after radiotherapy: state of the art and prevention [J]. Eur Ann Otorhinolaryngol Head Neck Dis.

[22] Ding J, Tu W, Hu H (2017). Design of Individualized Oral Radiotherapy Stent Based on 3D Printing Technique [J]. Zhongguo yi liao qi xie za zhi = Chinese J Med Instrumentation.

[23] Sciubba JJ, Goldenberg D (2006). Oral complications of radiotherapy [J]. Lancet Oncol.

